# Validity of Six Month L-Thyroxine Dose for Differentiation of Transient or Permanent Congenital Hypothyroidism

**DOI:** 10.4274/jcrpe.galenos.2020.2019.0170

**Published:** 2020-09-02

**Authors:** Muhammet Asena, Meliha Demiral, Edip Unal, Murat Öcal, Hüseyin Demirbilek, Mehmet Nuri Özbek

**Affiliations:** 1 Diyarbakır University of Health Sciences Turkey, Gazi Yaşargil Training and Research Hospital, Clinic of Paediatrics, Diyarbakır, Turkey; 2 Diyarbakır University of Health Sciences Turkey, Gazi Yaşargil Training and Research Hospital, Clinic of Paediatric Endocrinology, Diyarbakır, Turkey; 3 Hacettepe University Faculty of Medicine, Department of Paediatric Endocrinology, Ankara, Turkey

**Keywords:** Congenital hypothyroidism, transient, permanent, six month L-thyroxine dose

## Abstract

**Objective:**

The tendency to reduce thyroid stimulating hormone (TSH) referral cut-off values in congenital hypothyroidism (CH) neonatal screening programs has resulted in an increase in the incidence of CH, but also the referral of infants with mild transient elevation of TSH. Therefore, there is a need to develop markers for differentiation of transient elevated TSH and permanent CH as early as safely possible to avoid unnecessary treatment. The aim was to evaluate sixth-month L-thyroxine (LT4) dose as a predictive marker for differentiation of transient elevated TSH and permanent CH.

**Methods:**

Data of patients who had been followed after referral from the neonatal screening programme between the year 2010 and 2019 in a tertiary pediatric endocrine centre were examined retrospectively.

**Results:**

There were 226 cases referred, of whom 186 (82.3%) had eutopic thyroid gland, and 40 (17.7%) had dysgenetic gland. In patients with a dysgentic gland there was a non-significant tendency to have lower diagnostic free thyroxine concentration but significantly higher TSH compared with those with eutopic gland (p=0.44 and p=0.023, respectively). Patients with thyroid dysgenesis required higher initial and six month LT4 doses compared with those with eutopic glands (p=0.001). Receiver operator curve analysis showed the optimum cut-off value for LT4 at six months for transient vs. permanent CH was 2 μg/kg/day (sensitivity 77% and specificity 55%), regardless of etiology. Similarly, in patients with eutopic glands the optimum cut-off value for LT4 dose at six months for permanent vs. transient patients was 2 μg/kg/day (sensitivity 72% and specificity 54%).

**Conclusion:**

Results suggest that LT4 requirement at six months of therapy may be a good marker for predicting transient TSH elevation in patients with eutopic thyroid gland, thus facilitating the decision to halt LT4 therapy.

**What is already known on this topic?**

Setting a lower thyroid stimulating hormone (TSH) referral cut-off value in neonatal screening programs is increasingly common. However, this had resulted in referral of neonates with lower TSH concentrations and an increase in transient mild TSH elevation. Identifying infants who are likely to have transient TSH elevation and therefore withdrawing replacement therapy earlier would be of clinical benefit.

**What this study adds?**

L-thyroxine replacement dose requirement at the sixth month of therapy may be a good marker for predicting those with transient elevated TSH in patients with an eutopic thyroid gland.

## Introduction

Thyroid hormones are critical for normal brain development during the intrauterine period and early infancy. Therefore, delay in the diagnosis and treatment of congenital hypothyroidism (CH) will lead to severe neurological and psychiatric disorders (1,2). Indeed, CH is considered to be the most common cause of preventable mental retardation (1,3,4).

Neonatal screening for identification of infants suspected of having CH has improved the early diagnosis and immediate treatment of CH. The majority of screening programs use blood spot (capillary) thyroid stimulating hormone (TSH) concentration to identify infants requiring urgent assessment as central hypothyroidism accounts for only 2-5% of all neonates with CH (4,5). The referral cut-off value for TSH in neonatal screening programs has gradually declined from 20-50 µIU/mL at screening programme inception to lower values (1,4,6). Determination of lower cut off values has led to an increase in the incidence of CH from 1/3000-4000 (4), to 1/2000-3000 (1,7).

The number of referrals from screeing programmes has been increasing as the TSH cut-off values have reduced, while the incidence of permanent CH has not changed much (8,9). Therefore, developing criteria for differentiation of transient TSH elevation and permanent CH is important from the point of management. In this study, the clinical characteristics of the patients diagnosed with CH over a 10-year period were evaluated. The case characteristics were assessed to identify possible parameters that would help to differentiate the transient abnormalities and permanent CH at the diagnosis or during follow-up.

## Methods

Data of the patients who were being followed with the diagnosis of CH between January 2010 and January 2019 in Pediatric Endocrinology Outpatient Clinic of Health Sciences University, Diyarbakir Gazi Yasargil Training and Research Hospital were examined retrospectively. The study was performed in accordance with the Declaration of Helsinki and approved by the Institutional Ethics Committee of Gazi Yaşargil Training and Research Hospital (document number: 2019/334). Exclusion criteria were: patients diagnosed in another centre and were on LT4 replacement but with incomplete data about their initial LT4 dose and/or pretreatment thyroid function tests (TFTs); those lost to regular follow up; and those not yet three years of age.

The gender, age of diagnosis (in days), parental consanguinity, family history of thyroid disorders and complaints at presentation were recorded. The most recent TSH, free thyroxine (FT4), free tri-iodothyronine concentrations and height standard deviation (SD) score (SDS) and weight SDS, as well as LT4 doses of the patients at diagnosis, at the sixth month of the treatment, at the end of the treatment and during follow-up were noted. Results of thyroid imaging, including ultrasonography (USG) and 99mTc scintigraphy, were recorded.

**Figure 1:**
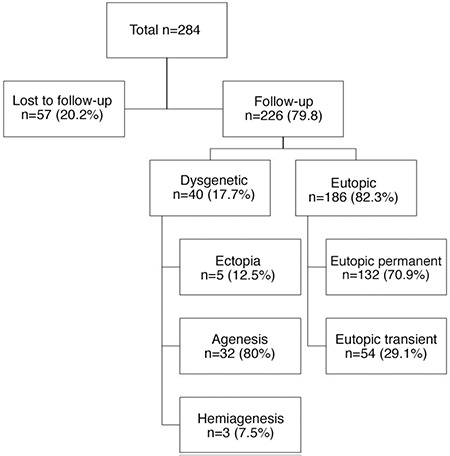
A flow-diagram of all patients with congenital hypothyroidism

Patients whose thyroid gland was of normal size and location on thyroid USG and/or thyroid scintigraphy were defined as eutopic CH. Thyroid dysgenesis was defined as cases with gland hypoplasia, ectopia, hemi-agenesis or complete agenesis (Dysgenetic CH). A trial of L-thyroxine (LT4) cessation was undertaken in all patients who had a eutopic thyroid gland, had reached the age of three years and no longer required dose increases due to continuing elevation of TSH, implying increasing LT4 requirement. TSH and FT4 were measured four weeks following cessation of LT4 treatment. Cases in whom TFTs were normal four weeks after cessation of LT4 and remained stable over the ensuing six months were considered to have transient eutopic CH. Cases who required reintroduction of LT4 replacement (TSH >10 µIU/mL) were defined as permanent eutopic CH (Figure 1) (8).

TSH and FT4 levels were analyzed on the Abbott Architect i8000 device (Abbott Park, Illinois, United States) using the electrochemiluminescence immunoassay “ECLIA” method. Normal range was considered to be 0.35-4.94 µIU/mL for TSH level and 0.70-1.48 ng/dL for FT4 level.

### Statistical Analysis

Statistical analyses were performed using Statistical Package for the Social Sciences for Windows, version 16 (IBM Inc., Chicago, IL, USA). Continuous variables were presented as mean±SD, or median (interquartile range), whereas categorical variables were presented as count and percentage (%). For evaluation of normality of distribution of the data, Shapiro-Wilk tests were used. For comparison of normally distributed data Student’s t-test was applied, whereas for comparison of non-normally distributed data Mann-Whitney U test were applied. A receiver operating characteristics (ROC) curve analysis was performed for determination of the best cut-off value for LT4 dose at the six month of the treatment between permanent and transient CH. A p value <0.05 was considered to indicate statistical significance.

## Results

In total, 284 patients diagnosed with CH within the first six months of life were eligible for inclusion. Fifty-eight patients were excluded from the study, as they were lost to follow-up (Figure 1). The remaining 226 patients (123 female) were included. The mean age of diagnosis was 41.18±39 (range 4-180) days. At first assessment, the mean TSH level was 81.79±35 mIU/mL and the mean FT4 level was 0.55±0.33 ng/dL. Starting L thyroxine dose was 7.04±2.64 µg/kg/day (Table 1). Etiological evaluation of cases revealed that 186 out of 226 (82.3%) cases were diagnosed with eutopic CH, whereas 40 (17.7%) were diagnosed with thyroid dysgenesis. Of the patients with eutopic CH; 132 (71%) had permanent and 54 (29%) had transient CH (Figure 1). The patients identified as transient TSH elevation with eutopic gland constituted 54/226 (23.9%).

While the FT4 concentrations measured at the time of the diagnosis were not statistically different between patients with eutopic and dysgenetic glands (p=0.44), TSH levels were significantly higher in cases with thyroid dysgenesis (p=0.023). Initial and sixth-month LT4 doses of cases with dysgenetic CH were significantly higher compared to eutopic CH patients (p=0.001) (Table 2).

When cases of transient and permanent CH were compared, no difference was determined in any parameter except for the LT4 dose at the sixth month and discontinuation of the treatment (Table 2).

**Figure 2:**
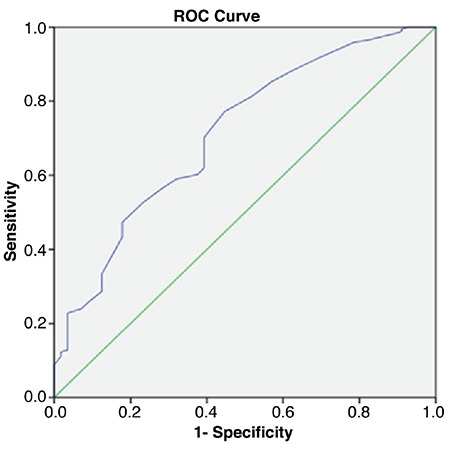
The receiver operating characteristics analysis for L-thyroxine dose at the 6th month for eutopic transient vs. permanent congenital hypothyroidism ROC: receiver operating characteristics

Of the permanent cases, 54 (58%) had eutopic CH and 40 (42%) had dysgenetic CH. The ratio of consanguinity in permanent cases was similar in both groups (55.5%). LT4 dose at the sixth month was higher in cases with permanent CH (2.92±1.2 µg/kg) then in those with transient CH (2.13±0.88 µg/kg) (p<0.001) (Table 2). Doses at the sixth month were higher in dysgenetic cases compared to the eutopic cases (3.26±1.1 µg/kg; 2.60±1.18 µg/kg, respectively) (p=0.001). In the ROC analysis, regardless of the aetiology, the optimum cut-off value for LT4 dose at the sixth month for transient vs. permanent CH was 2 µg/kg/day [area under curve (AUC): 0.713; sensitivity 77%; specificity 55%; p<0.001]. The optimum cut-off value for LT4 dose at the sixth month for eutopic permanent vs. eutopic transient CH was also 2 µg/kg/day (AUC: 0.677; sensitivity 72%; specificity 54%; p<0.001). The positive predictive value of the treatment dose of 2 µg/kg/day at six months in patients with eutopic CH was 59.2% (Figure 2).

## Discussion

In the present study 226 patients diagnosed with CH were evaluated and a high rate of transient cases was observed. The sixth-month LT4 doses were lower in transient cases and it is suggested that this parameter may function as a marker for differentiating permanent and transient CH.

Although a nation-wide neonatal screening program is available in Turkey, the age of presentation of our cases (44.18±39 days) was a bit late than expected. This was attributed to the fact that the majority of patients were from rural areas and probably have some difficulties in accessing their results of screening as well as in admission to our tertiary pediatric endocrine centre. While in some studies, a female predominance has been reported, some others, showed a male predominance (8,9,10,11,12,13,14). In the present study, there was a slight female predominance.

Regarding the aetiology of CH, 186 out of 226 cases (82.4%) had eutopic thyroid gland. Of which 132 (71%) cases had permanent CH. Patients with eutopic thyroid gland account for (54%) of cases with permanent CH (Figure 1). When considering the aetiology of permanent CH, in previous studies, thyroid dysgenesis was reported to cause 75-85% of permanent CH cases which was followed by dyshormonogenesis cases with eutopic glands (5,13). However, a considerable shift has been observed in the incidence rate as well as the ratio of transient and permanent cases with a change in the cut-off values which had been used for neonatal screening programs (5,8,15). In our series an increase in the number of cases with transient hypothyroidism can be attributed to moderate iodine deficiency, while the high rate of permanent cases may be attributed to the high rate of consanguinity (55%) which may be associated with increased risk of autosomal recessive dyshormonogenesis (5,16,17,18).

Prompt and correct diagnosis and differential diagnosis of cases admitted from neonatal screening program are critically important for avoiding missing cases as well as introducing the most appropriate treatment. Indeed, unnecessary and overtreatment may associate with an economic burden and poor neurodevelopmental outcome (3,19). Nevertheless, the presenting hormonal features are usually overlapping and do not allow to make a differential diagnosis of transient and permanent CH. In keeping with this, transient CH accounts for 55% of our cases and cases with transient and permanent CH did not have statistically significant different TSH and FT4 levels (Table 2). There was also no difference between permanent and transient CH in cases with a eutopic thyroid gland. However, when dysgenetic and eutopic CH were compared, dysgenetic cases had higher mean TSH and lower mean FT4 levels (Table 2). Although this data may help to estimate dysgenetic cases, this is not a reliable method as these values may overlap frequently. There are also studies reporting that TSH level at assessment is higher in patients with permanent CH compared to transient CH (20).

Evaluation of LT4 doses in dysgenetic/eutopic, total transient/permanent and eutopic transient/eutopic permanent CH, revealed a significant difference in doses required at the age of six months (Table 2). ROC analysis revealed an optimal cut-off value of 2 µg/kg/day for LT4 dose at the sixth month, for differentiation of cases with eutopic transient and eutopic permanent CH, with a sensitivity of 72% and a specificity of 54% (Figure 2). Similarly, Oron et al (21) have reported a cut-off value for sixth-month LT4 dose of 2.2 µg/kg in retrospective analysis of 142 cases. However, in the study of Saba et al (22) the cut-off value for treatment doses at the sixth month of 49 transient and 43 permanent CH patients with a eutopic thyroid gland was reported as 3.2 µgr/kg/day, with a sensitivity of 71% and a specificity of 79%. The mean time of discontinuation of treatment of transient cases was 1.5 years of age. The treatment dose at the sixth month was higher than the cut-off value we reported for our eutopic transient and permanent CH patients. For our cases, mean FT4 at the sixth month was 1.28±0.51 and the mean time of discontinuation of treatment in eutopic transient cases was 26 months. The discrepancy between the cut off values in our and Saba’s study could be attributed to higher LT4 doses they have used in their patients.

In a multi-centre retrospective study evaluating LT4 doses of cases with CH for 12 years, LT4 doses received per kg of body weight was shown to gradually decrease every year, starting from the sixth month of age (21). By the 12th year, while eutopic CH cases were receiving a mean dose of 1.7 µg/kg/day, patients with ectopic gland and agenesis required a dose of 2.1 and 2.2 µg/kg/day, respectively, with no statistically significant difference (21). LT4 dose at the sixth month was lower in cases with eutopic CH compared to the other two groups. In the study by Unüvar et al (10) in which they compared cases with permanent CH and hyperthyrotropinemia, they did not observe any difference between the groups other than the required LT4 dose at the 1st year (4.79±2.09 and 3.46±1.23 µg/kg/day, respectively). In a study of 204-case series from a neighbouring country, Iran, a statistically significant difference was reported in only total LT4 doses between transient and permanent CH (40.0 + 12.77 µg/day and 48.3 + 47.64 µg/day, respectively) (9).

Messina et al (23) reported that LT4 doses at 1^st^, 2^nd^ and 3^rd^ years were predictive in early differentiation of transient and permanent CH. They reported that predictive cut-off values for transient eutopic CH were 1.7 µg/kg/day, 1.45 µg/kg/day and 0.98 µg/kg/day, respectively with a sensitivity of 100%. However, they did not report the dose at the sixth month.

There are limitation of present study. Firstly, as we recruited cases retrospectively we could not access and take into account the birth weight and clinical status of cases. We also have not data about maternal and fetal iodine status as well as breastfeeding status. In addition, in our laboratory the upper detectable limit for TSH were 100 µIU/mL and TSH levels above 100 µIU/mL was noted as 100 µIU/mL in patients’ hospital records. This might caused an underestimation and overlap of TSH levels between TSH levels of cases.

## Conclusion

In conclusion, setting a lower TSH level as cut-off for neonatal screening programs is increasingly common. This has resulted in diagnosing more transient elevated neonatal TSH and mild hypothyroidism. There is therefore a need to investigate criteria which may identify babies in which it is safe to stop replacement thyroxine therapy earlier. LT4 doses required at the sixth month may be a useable marker for predicting transient-permanent eutopic CH patients. As a delay in discontinuation of treatment and overtreatment may be associated with worse neurological outcome, increased anxiety for both families and physicians, as well as for insurance systems, the results presented here may contribute to an earlier clinical decision regarding discontinuation of therapy. Larger studies adjusting for iodine status of the population, breast feeding status of the included infants and clear diagnostic criteria for dysgenetic, dyshormonogenetic and other babies with gland *in situ* are warranted to investigate if evaluating LT4 doses required at the sixth month is safe and sensitive enough to distinguish cases with transient and permanent eutopic CH.

## Figures and Tables

**Table 1:**
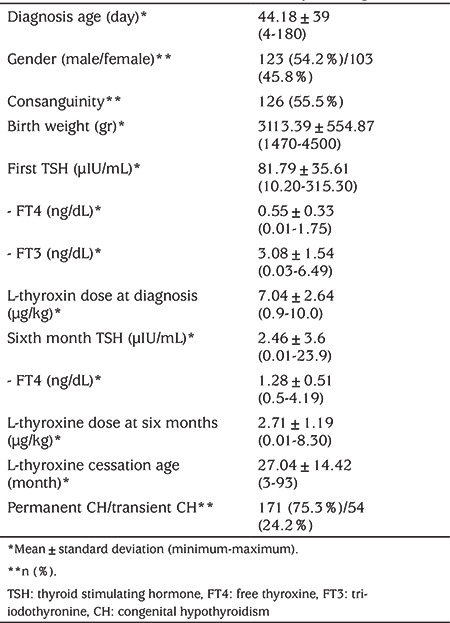
Patient’s clinical and laboratory findings

**Table 2:**
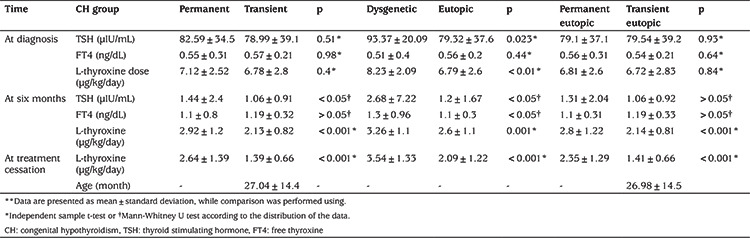
Comparisons between groups parameters**
